# Identification of the Key Genes Associated with Different Hair Types in the Inner Mongolia Cashmere Goat

**DOI:** 10.3390/ani12111456

**Published:** 2022-06-04

**Authors:** Gao Gong, Yixing Fan, Wenze Li, Xiaochun Yan, Xiaomin Yan, Ludan Zhang, Na Wang, Oljibilig Chen, Yanjun Zhang, Ruijun Wang, Zhihong Liu, Wei Jiang, Jinquan Li, Zhiying Wang, Qi Lv, Rui Su

**Affiliations:** 1 College of Animal Science, Inner Mongolia Agricultural University, Hohhot 010018, China; ggao1995@163.com (G.G.); 18394317062@163.com (W.L.); yanxiaochunan@163.com (X.Y.); 18648669958@163.com (X.Y.); zld18181508061@163.com (L.Z.); imauzyj@163.com (Y.Z.); nmgwrj@126.com (R.W.); liuzh7799@163.com (Z.L.); jiangwei1022@163.com (W.J.); lijinquan_nd@126.com (J.L.); wzhy0321@126.com (Z.W.); 2 College of Animal Science and Veterinary Medicine, Shenyang Agricultural University, Shenyang 110866, China; fanyx@syau.edu.cn; 3 Inner Mongolia Yiwei White Cashmere Goat Co., Ltd., Hohhot 010018, China; 13947377140@163.com (N.W.); w15047776757@163.com (O.C.); 4 Key Laboratory of Animal Genetics, Breeding and Reproduction, Inner Mongolia Agricultural University, Hohhot 010018, China; 5 Key Laboratory of Mutton Sheep Genetics and Breeding, Ministry of Agriculture and Rural Affairs, Hohhot 010018, China; 6 Engineering Research Center for Goat Genetics and Breeding, Inner Mongolia Agricultural University, Hohhot 010018, China

**Keywords:** different hair types, Inner Mongolia cashmere goats, hair growth, WGCNA, KRT

## Abstract

**Simple Summary:**

The Inner Mongolia cashmere goat is an excellent local breed in China. According to the characteristics of wool quilts, the Inner Mongolia cashmere goat can be divided into three types: a long-hair type (hair length of >22 cm), a short-hair type (hair length of ≤13 cm), and an intermediate type (hair length of >13 cm and ≤22 cm). In order to explore the molecular mechanisms and related regulatory genes of the different hair types, a weighted gene coexpression network analysis (WGCNA) was carried out on the gene expression and phenotypic data of 12-month-old Inner Mongolia cashmere goats of the long-hair (LHG) and a short-hair type (SHG). There is a strong correlation between one module and different hair types. The expression trends of this module’s genes were different between the LHG and SHG. The function of GO is mainly concentrated in cellular components, including intermediate filaments, intermediate filament cytoskeletons, and cytoskeletons. *KRT39*, *KRT74*, *LOC100861184*, *LOC102177231*, *LOC102178767*, *LOC102179881*, *LOC106503203*, *LOC108638293*, and *LOC108638298* were significantly different between the two hair types, and most of them were positively correlated with hair length. It is speculated that these candidate genes can regulate different hair types of cashmere goats and provide molecular markers for the hair growth of cashmere goats.

**Abstract:**

The Inner Mongolia cashmere goat is an excellent local breed in China. According to the characteristics of wool quilts, the Inner Mongolia cashmere goat can be divided into three types: a long-hair type (hair length of >22 cm), a short-hair type (hair length of ≤13 cm), and an intermediate type (hair length of >13 cm and ≤22 cm). It is found that hair length has a certain reference value for the indirect selection of other important economic traits of cashmere. In order to explore the molecular mechanisms and related regulatory genes of the different hair types, a weighted gene coexpression network analysis (WGCNA) was carried out on the gene expression data and phenotypic data of 12-month-old Inner Mongolia cashmere goats with a long-hair type (LHG) and a short-hair type (SHG) to explore the coexpression modules related to different coat types and nine candidate genes, and detect the relative expression of key candidate genes. The results showed that the WGCNA divided these genes into 19 coexpression modules and found that there was a strong correlation between one module and different hair types. The expression trends of this module’s genes were different in the two hair types, with high expression in the LHG and low expression in the SHG. GO functions are mainly concentrated in cellular components, including intermediate filaments (GO:0005882), intermediate filament cytoskeletons (GO:0045111), and cytoskeletal parts (GO:0044430). The KEGG pathway is mainly enriched in arginine as well as proline metabolism (chx00330) and the MAPK signaling pathway (chx04010). The candidate genes of the different hair types, including the *KRT39*, *KRT74*, *LOC100861184*, *LOC102177231*, *LOC102178767*, *LOC102179881*, *LOC106503203*, *LOC108638293*, and *LOC108638298* genes, were screened. Through qRT-PCR, it was found that there were significant differences in these candidate genes between the two hair types, and most of them had a significant positive correlation with hair length. It was preliminarily inferred that these candidate genes could regulate the different hair types of cashmere goats and provide molecular markers for hair growth.

## 1. Introduction

Cashmere goats are excellent breeds for the dual purpose of cashmere and meat. Cashmere has become a precious textile raw material because of the advantages of soft fibers, good glossiness, and warm, lightweight, and comfortable products, and it is known as “soft gold” and “fiber gem.” Cashmere goats are mainly distributed in parts of Eurasia in the Northern Hemisphere, although a small number of cashmere goats are also distributed in the Southern Hemisphere, in Australia and New Zealand [1,2,3]. The coat of cashmere goats is typically heterogeneous fleece. In the skin, the primary hair follicle (PHF) produces myelinated coarse hair, and the secondary hair follicle (SHF) produces unmyelinated cashmere. Inner Mongolia cashmere goats belong to seasonal depilation animals, and the growth of cashmere has a typical periodic law, which can be divided into three periods, including anagen (April to November), catagen (December to January of the following year), and telogen (February to March) [4,5,6].

As a tiny organ of hair growth, hair follicles are regulated by many factors, such as heredity, nutrition, season, age, shearing, environment, and so on [6,7,8,9]. A number of studies have shown that a variety of signal pathways are involved in regulating the growth, development, and periodic changes of cashmere goat hair follicles, including the Wnt signaling pathway, the TGF-beta signaling pathway, the MAPK signaling pathway, the Notch signaling pathway, and other signal pathways [10,11]. Fibroblast growth factors (FGFs), keratins (KRTs), keratin-associated proteins (KRTAPs), and other molecules also play a regulatory role in the growth and development of hair follicles in cashmere goats [12,13]. Hair growth is also affected by a variety of hormones. Prolactin and melatonin have been found to affect the growth and activity of hair follicles, which can directly stimulate the elongation of the hair stem of secondary hair follicles [14,15].

**Figure 1:**
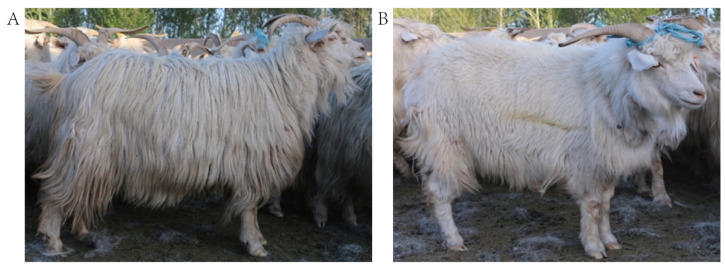
Photographs of long-hair-type and short-hair-type cashmere goats. (**A**) Long-hair-type cashmere goat, (**B**) short-hair-type cashmere goat.

There are some disadvantages in the detection of goat cashmere quality, such as its high labor intensity and it being a time-consuming process, but it is easy for the length of hair to be observed and identified. JQ Li and other authors have found that hair and cashmere have strong phenotypic and genetic correlation. Hair length and cashmere length showed a high positive genetic correlation (0.51); hair length and cashmere fineness showed a negative genetic correlation (−0.28) [16]. Therefore, wool length has a certain reference value for the indirect selection of cashmere quality traits, which is helpful to accelerate the progress of the genetic improvement of cashmere goats. The research on the diversity of coarse wool fiber traits is abundant in the production and breeding of Chinese cashmere goats. Using a statistical method, it was found that there are great differences in hair length among different individual Inner Mongolia cashmere goats, and the range of variation is between 5 and 34 cm. The hair can be divided into three types according to its length: a long-hair type (hair length of >22 cm), a short-hair type (hair length of ≤13 cm), and an intermediate type (hair length of >13 cm and ≤22 cm), which provide a basis for the classification of different hair types. Through phenotypic and genetic correlation analyses, it was found that there was a very significant correlation between hair length and other cashmere quality traits (cashmere thickness, cashmere yield, cashmere weight, cashmere length, hair fineness, and cashmere fineness) (*p* < 0.01) [17]. Among them, the typical long-hair type has the characteristics of long and bright hair as well as long and white cashmere, while the typical short-hair type has the characteristics of rough hair, sparse hair, and poor luster (Figure 1). Some studies have found that hair fineness and cashmere fineness decrease with an increase in hair length, and that cashmere length increases with an increase in hair length. Among the three hair types, the long-hair type has the finest hair and cashmere fineness, as well as the longest cashmere length. Long-hair-type individuals have the highest phenotypic value of cashmere yield and body weight, the highest heritability, and the greatest genetic correlation. The selection of long-hair-type individuals can indirectly select other cashmere quality traits, such as body weight, cashmere yield, and cashmere fineness, so as to realize the indirect selection of other important economic traits [18,19].

The WGCNA method is an excellent tool for analyzing the important economic traits of livestock and poultry through sequencing data, and has been widely used in livestock and poultry research [20,21,22]. At present, the genetic law of different quilt types of Inner Mongolia cashmere goats is not clear. In this study, the transcriptome sequencing data of different hair types were used in the laboratory. They were analyzed by the method of a WGCNA. The purpose of this study was to find the key regulatory factors regulating different hair types and to provide new molecular markers for hair growth and hair development related research.

## 2. Materials and Methods

### 2.1. Data Sources

The data of this study are derived from the results of the transcriptome of Inner Mongolia cashmere goats with different hair types. The samples were 3 long-hair cashmere goats (LHGs) and 3 short-hair cashmere goats (SHGs) of 2-year-old Inner Mongolia cashmere goats (Alba type). From July 2016 to June 2017, 72 samples were collected from the skin tissue on the 20th of each month. Extraction of RNA from skin tissue: RNA-seq was carried out by using an Illumina X Ten sequencing platform system. Sequence alignment was carried out with goat genome (Capra hircus, ARS1) by HISAT (2.0.4) [23]. The gene expression level of each sample was analyzed by HTSeq (v0.6.1) [24], and the gene expression data (FPKM) were calculated. The genes with zero expression in more than 16 samples were filtered, the differential expression of genes between the two groups was analyzed by DESeq (padj < 0.05) [25], and all of the differential genes were merged. A total of 7320 transcripts were obtained by filtering. Among them, the January skin samples of long-hair cashmere goats were marked with LHG_1(_1,_2,_3), and the January skin samples of short-hair cashmere goats were marked with SHG_1(_1,_2,_3). At the same time, the sequenced individuals included the phenotypic data of 6 traits: cashmere fine, hair fine, cashmere yield, cashmere length, hair length, and hair type.

### 2.2. Construction of Weighted Gene Coexpression Network

In this study, the WGCNA package in R(3.2.5) (R, The R Foundation) was used for analysis (refer to the official WGCNA website tutorial (https://horvath.genetics.ucla.edu/html/CoexpressionNetwork/Rpackages/WGCNA/) (accessed on 20 March 2020) [26,27] to download the R package) and to construct a gene coexpression network based on the gene expression data and phenotypic matrix of the samples. First of all, the samples are analyzed by a cluster analysis; the outliers in the samples are removed, as the outliers will interfere with the analysis of the network module. Following this, through the construction of three matrices for a follow-up analysis, the network constructed with a soft threshold parameter (β) conforms to the standard scale-free network as much as possible, and retains connectivity information, which is also consistent with the relationship between biological genes. We choose the minimum β when R^2^ > 0.8 to build the network. The topological overlap matrix (TOM) clustering tree is drawn, and the genes with similar expression patterns are clustered into one group through the topological tree structure. The TOM values between genes are used for hierarchical clustering, and the modules are divided and merged. Different gene modules are represented by different colors, and the same color means that all genes in that color are divided into the same module. The genes in these modules may have similar expression changes in a physiological process or in different tissues. Accordingly, these genes may have the same function.

### 2.3. Screening of Candidate Modules

A module correlation analysis was carried out through the correlation between phenotypic trait information and gene module eigenvalues. A Pearson correlation analysis was used to calculate the correlation coefficient and *p*-value between gene module eigenvalues and phenotypic trait data information, and a module–trait correlation heat map was constructed to determine the modules associated with different hair types. The module with the highest correlation coefficient with the traits was selected as the target module, and the gene information in the target module was further analyzed.

### 2.4. Enrichment Analysis of Candidate Modules

Candidate modules were selected. By calculating the expression patterns of module eigenvalues in different samples, a heatmap of genes’ expression patterns was drawn. The genes in the target module were analyzed by Gene ontology (GO) functional enrichment analysis and Kyoto Encyclopedia of Genes and Genomes (KEGG) pathway enrichment analysis to explore the regulatory function and role of module genes in different hair types [28,29]. The GO enrichment analysis of differentially expressed genes was implemented by the GOseq [30] R package, in which the gene length bias was corrected. GO terms with a corrected *p*-value less than 0.05 were considered significantly enriched by differential expressed genes. We used the KOBAS [31] software (KOBAS Software, Beijing, China) to test the statistical enrichment of differential expression genes in KEGG pathways. The enrichment was considered to be significant when the corrected *p*-values were less than 0.05.

### 2.5. Candidate Gene Screening

The exportNetworkToCytoscape function in the WGCNA was used to derive the network relationship (threshold = 0.10). The output module nodes file and the module edges file were imported into the Cytoscape 3.9.0 (Cytoscape Software, CA, USA) [32] for network visualization. According to the results of gene functional enrichment and the gene connectivity network, some candidate genes were selected, and the FPKM value was used to draw the gene expression trend map.

### 2.6. Sample Collection

In this study, skin samples were collected in accordance with the International Guiding Principles for Biomedical Research Involving Animals and were approved by the Animal Ethics Committee of the Inner Mongolia Academy of Agriculture and Animal Husbandry Sciences, which is responsible for animal care and use in the Inner Mongolia autonomous region of China. The experimental animals came from the Inner Mongolia Jinlai Livestock Technology Company (Hohhot, Inner Mongolia, China). According to the production performance records and hair length data in 2018, 7 adult Inner Mongolia cashmere goats with a similar LHG (hair length was 23~26 cm) and SHG (hair length was 7~13 cm) were selected. Skin tissue of 1 cm^2^ in the metacarpal behind the scapula was collected in September (anagen), December (catagen), and March of the following year (telogen). The skin tissue was immediately placed in liquid nitrogen and then stored in a cryogenic refrigerator at −80 °C for the subsequent extraction of total RNA.

### 2.7. Quantitative Real-Time PCR (qRT-PCR)

The total RNA was extracted using an RNA extraction reagent (Trizol Reagent, Invitrogen, Waltham, MA, USA) by following the user guide. The concentration and quality of the total RNA were determined by a NanoDrop 2000 (Thermo, Waltham, MA, USA). The quality of the RNA was detected by 1.0% agarose gel electrophoresis and photographed by a gel imager. The qualified total RNA was synthesized by a reverse transcription kit to synthesize cDNA (PrimeScript™ RT reagent Kit with gDNA Eraser (Perfect Real Time), RR047A, TAKARA, Kusatsu City, Japan). In reference to the manual, it was removed after the reaction was completed and stored in a refrigerator at −80 °C.

Referring to the mRNA coding region sequence of goat genes in the NCBI database, the fluorescent quantitative specific primers of goat genes were designed by using Primer-BLAST [33] in the NCBI database. The primer parameters were set as follows: PCR product size, 100–300 bp; primer size, 18~24 bp; primer GC content, 40–60%; and other parameters as default values. All of the primers were handed over to Sangon Biotech (Shanghai) Co., Ltd. (Shanghai, China), and the sequence of the primers is shown in Table 1.

The qRT-PCR test was carried out by a fluorescence quantitative kit (TB Green™Premix Ex Taq™ II (Tli RNaseH Plus), RR820A, TAKARA, Kusatsu City, Japan) and a LightCycler ^®^96 (Roche, Basel, Switzerland). The reaction system was TB Green Premix Ex Taq II (Tli RNaseH Plus) (2X), 5 μL; cDNA, 1 μL; PCR forward/reverse primer (10 μM), 0.5 μL; and ddH_2_O 3 μL. Programs: preincubation at 95 °C for 30 s, 2-step amplification (95 °C 5 s, Tm 30 s) for a total of 45 cycles, melting, and cooling. There were 7 biological repeats in each group, and 3 technical repeats were performed in each sample. The relative expression of the genes was calculated by 2^−ΔΔ^CT [34]. At the same time, the ratio of the differential multiple (R) of gene expression between LHGs and SHGs was calculated. 

The specific calculation formula is as follows: double house-keeping genes, *Cq*, value calculation formula: $Cq_{\mathrm{house}-\mathrm{keeping}\;\mathrm{genes}}=\sqrt{Cq_{GAPDH}\times Cq_{\beta -actin}}$. The ratio of the difference multiple: $R=F_{\mathrm{LHG}}/F_{\mathrm{SHG}}$.

### 2.8. Data Analysis

Excel 2019 was used to collate and summarize the data, SAS 9.2 ANOVA (SAS Institute, Inc., Cary, NC, USA) was used to analyze the variance of the data, SAS 9.2 CORR (SAS Institute, Inc., Cary, NC, USA) was used to analyze the correlation of the data, and GraphPad Prism 8.3.0 (GraphPad Software, San Diego, CA, USA) was used to draw the relative gene expression map. *p* < 0.05 means a significant difference, and *p* < 0.01 means an extremely significant difference.

## 3. Results

### 3.1. Construction of Weighted Gene Coexpression Network

**Figure 2:**
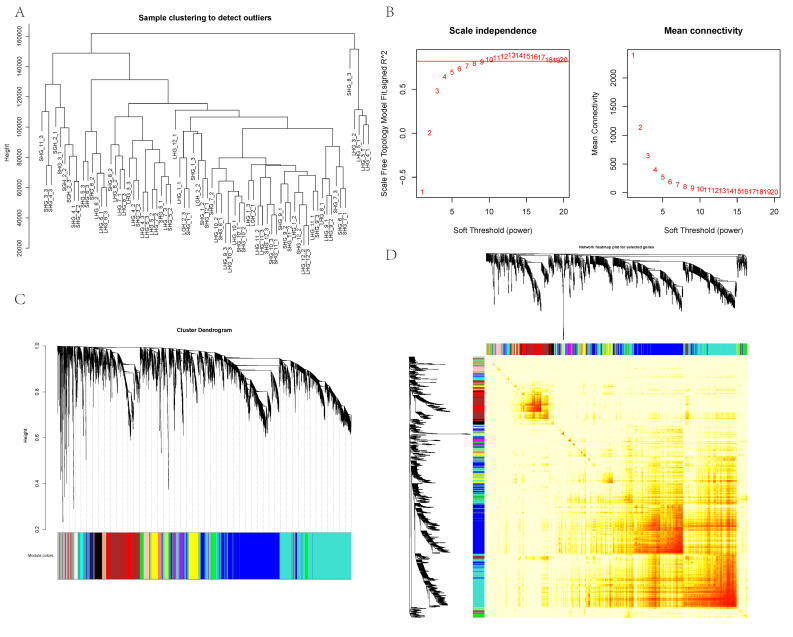
Construction of the weighted gene coexpression network. (**A**) Hierarchical clustering information of samples, (**B**) soft threshold filtering, the red line is β = 0.8, and the red number represents the β value, (**C**) gene coexpression network gene clustering number, and (**D**) network heatmap of gene–gene, different colors represent different gene modules.

From the 72 samples, 7320 transcripts were screened by transcriptome gene expression data, which were used to construct a weighted gene coexpression network. Through the hierarchical clustering of samples, it was found that the distribution of samples was more uniform and that there were no obvious outliers (Figure 2A). In order to construct a network that conformed to the scale-free distribution and retained the data information as much as possible, the best β value was found by the method of a soft threshold (Figure 2B). It is found that, when β = 10, the connectivity between genes in the network was high. Hierarchical clustering was carried out by using the TOM value between genes, and the modules were divided. The genes were divided into 19 modules by gene module clustering (Figure 2C). The statistical details of the number of genes in each module are shown in Table 2. Each module is represented by different colors, in which the number of genes aggregated by the turquoise module was the highest (2207), while that of the light-green module was the lowest (48). Subsequently, a gene–gene clustering heat map (Figure 2D) was constructed, and it was found that there was a strong correlation among genes in modules such as red, magenta, midnight blue, and turquoise.

### 3.2. Key Modules Related to Hair Follicle Cycle Development 

**Figure 3:**
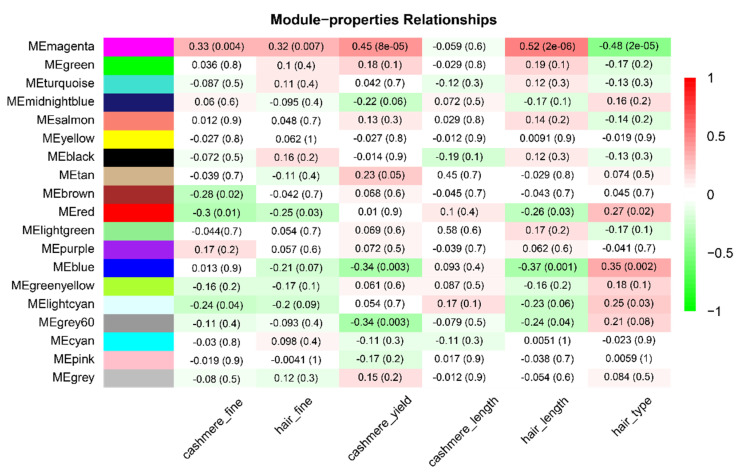
Module–properties relationships. Abscissa is the properties and the ordinate is the module name. The number in the grid indicates the Pearson correlation coefficient between the module and the character. The closer the value is to ± 1, the stronger the correlation between the module and the character is, and the number in parentheses represents a significant *p*-value. The Pearson correlation coefficient judged the correlation between module genes and phenotypic traits, and the *p*-value judged the significant degree of correlation.

The module and phenotypic traits are used to construct a module–properties relationship heatmap (Figure 3), which can reflect the correlation between modules and traits, so as to screen out important candidate modules. By observing the absolute value of the correlation between the modules and traits, it was found that the magenta module and the blue module had the highest correlations with the different hair types, which were −0.48 and 0.35, respectively. These two modules may be involved in the biological processes related to hair growth and development. The magenta module contains 128 genes. The magenta module has a strong correlation with different hair types (−0.48, *p* = 2 × 10^−5^), and this module has a strong correlation with cashmere fineness (0.33, *p* = 0.004), hair fineness (0.32, *p* = 0.007), cashmere yield (0.45, *p* = 8 × 10^−5^), and hair length (0.52, *p* = 2 × 10^−6^). It is predicted that the genes in this module may be strongly associated with the different hair types. The blue module contains 1839 genes. The correlation between the blue module genes and different hair types was 0.35 (*p* = 0.002), and there was also a certain correlation with cashmere yield (−0.34, *p* = 0.003) and hair length (−0.37, *p* = 0.001). Because the magenta module has a high correlation and significance in different hair types, cashmere yield, and hair length, the later stage focuses on the regulation of the magenta module on different hair types.

### 3.3. Magenta Module Function Analysis

**Figure 4:**
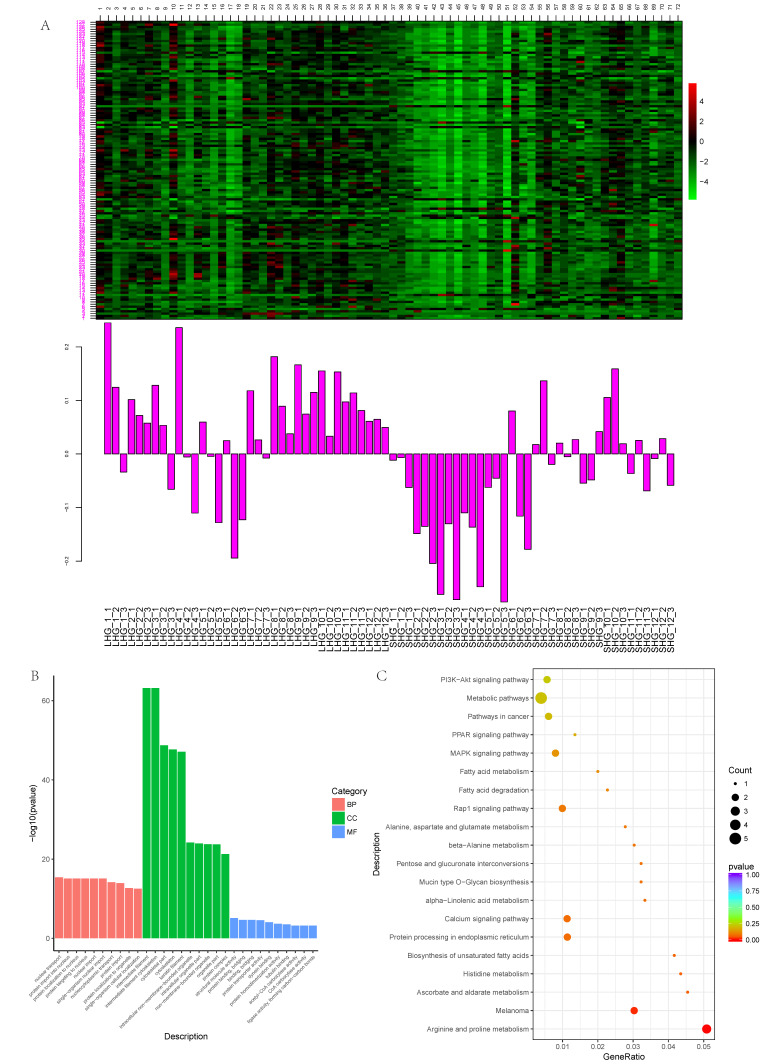
Magenta module function analysis. (**A**) Heatmap of the magenta module’s gene expression patterns, (**B**) GO analysis of the magenta module, and (**C**) KEGG enrichment analysis of the magenta module.

The magenta module contains 128 genes. The detailed gene information is shown in Supplemental Table S1. Through the heatmap of the magenta module’s gene expression patterns (Figure 4A), it was found that there was a difference in gene expression between LHGs and SHGs in this module. It was found that the expression trend of this module’s genes was significantly different between LHG and SHG individuals: the expression of LHG samples was generally high in this module, while that of SHG samples was generally low. It can also be seen that there are differences in the genes of this module from January to June and from July to December as a whole. The genes in the magenta module were analyzed by GO functional enrichment analysis (Figure 4B, Supplemental Table S2). It was found that 36 biological processes, 26 cellular components, and 11 molecular functions were significantly enriched. The biological processes are mainly concentrated in nuclear transport (GO:0051169), protein import into the nucleus (GO:0006606), protein localization to the nucleus (GO:0034504), protein targeting to the nucleus (GO:0044744), nuclear import (GO:0051170), single-organism nuclear import (GO:1902593), and so on. Cell components are mainly enriched in intermediate filaments (GO:0005882), intermediate filament cytoskeletons (GO:0045111), cytoskeletal parts (GO:0044430), cytoskeletons (GO:0005856), keratin filaments (GO:0045095), intracellular non-membrane-bounded organelles (GO:0043232), and other items, and there are a large number of genes in these items. The molecular functions are mainly concentrated in structural molecule activity (GO:0005198), protein binding, bridging (GO:0030674), binding, bridging (GO:0060090), protein transporter activity (GO:0008565), dynein binding (GO:0045502), and so on. The KEGG pathway analysis (Figure 4C, Supplemental Table S3) showed that the module was not only significantly enriched in arginine and proline metabolism (chx00330), but also enriched in melanoma (chx05218), the MAPK signaling pathway (chx04010), ascorbate as well as aldarate metabolism (chx00053), and the biosynthesis of unsaturated fatty acids (chx01040).

### 3.4. Screening of Candidate Genes for Different Hair Types

**Figure 5:**
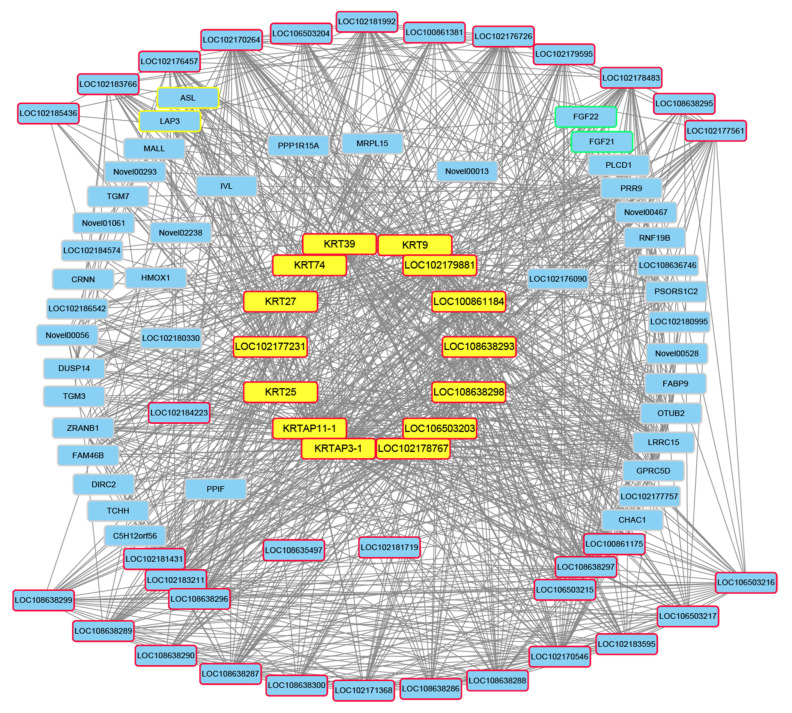
Gene coexpression network in the magenta module. The genes framed in red are the genes of the intermediate filaments (GO:0005882), the genes framed in yellow are arginine and proline metabolism (chx00330), the genes framed in green are the MAPK signaling pathway (chx04010), and the genes with a yellow background are the candidate genes.

In order to further screen the candidate genes, genes with high gene connectivity were selected and combined with the results of the GO and KEGG analyses, after which a gene network diagram was drawn by Cytoscape software (Figure 5). It was found that the genes in the GO entry intermediate filament (GO:0005882) had strong connectivity in the network. According to the results of a comprehensive analysis, some candidate genes related to different quilt types were selected, and the details are shown in Table 3, including *KRT9*, *KRT25*, *KRT27*, *KRT39*, *KRT74*, *KRTAP3-1*, *KRTAP11-1*, *LOC100861184*, *LOC102177231*, *LOC102178767*, *LOC102179881*, *LOC106503203*, *LOC108638293*, and *LOC108638298*.

**Figure 6:**
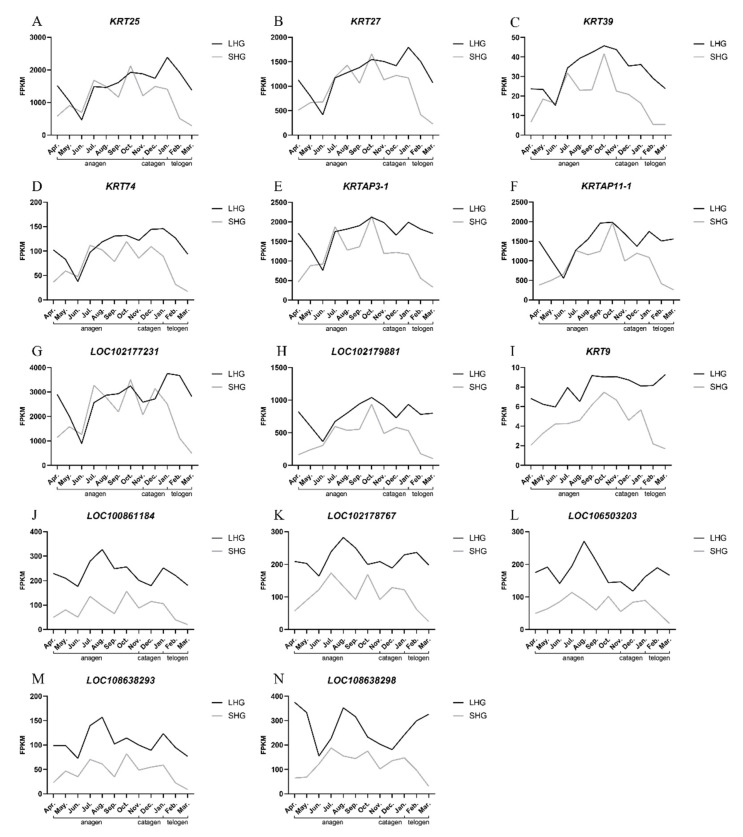
Gene expression trends of the candidate genes. (**A**–**N**) Abscissa is month, anagen (April to November), catagen (December to January), and telogen (February to March); ordinate is the FPKM.

The FPKM values of the genes were used to draw the expression trend maps of the candidate genes (Figure 6). By observing the expression trend map of each gene, it was found that the expression level of LHGs was higher than that of SHGs among the 14 candidate genes. It was found that the expression trends of the eight genes *KRT25*, *KRT27*, *KRT39*, *KRT74*, *KRTAP3-1*, *KRTAP11-1*, *LOC102177231*, and *LOC102179881* were basically the same in the two hair types. During the whole anagen, the gene expressions of LHGs and SHGs were basically the same, the difference was not obvious, and the overall expressions showed a gradual upward trend. There were significant differences between the two hair types during catagen and telogen. The gene expression level of LHGs was higher than that of SHGs, and the gene expression of SHGs decreased rapidly in catagen and telogen. Among them, the expression trends of the *KRT9*, *LOC100861184*, *LOC102178767*, *LOC106503203*, *LOC108638293*, and *LOC108638298* genes in the two hair types were basically the same. In the whole hair growth cycle, the expression of LHGs was higher than that of SHGs, and the expression trends of the two hair types were significantly different in the middle of anagen and telogen.

### 3.5. Detection of Relative Expression of Candidate Genes by qRT-PCR

**Figure 7:**
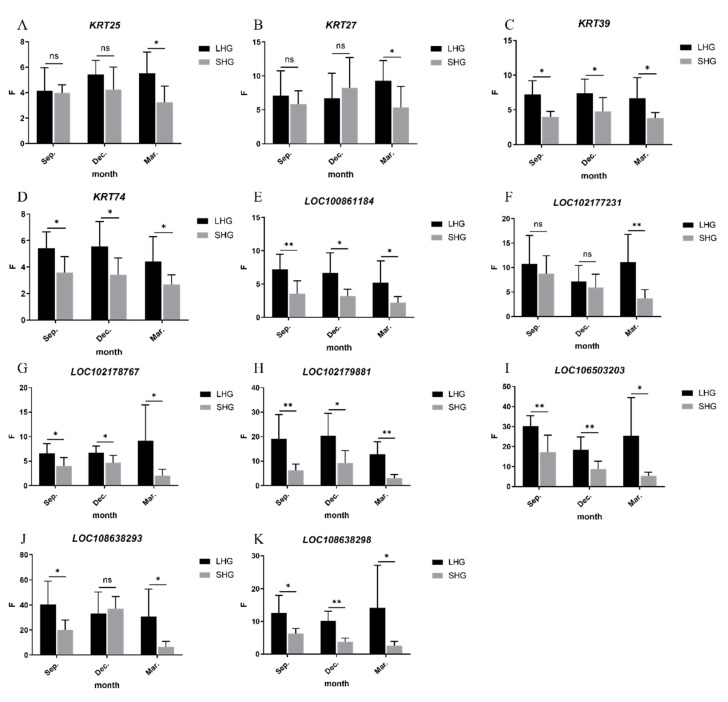
The relative expressions of the candidate genes. (**A**–**K**) Abscissa indicates period, anagen (September), catagen (December), and telogen (March); ordinate was relative expression quantity F = 2^−ΔΔCT^, ** indicates extremely significant difference, * indicates significant difference, and ns indicates no significant difference.

The total RNA was extracted from 42 skin samples, and the quality of the total RNA extraction affected the accuracy of the qRT-PCR results. NanoDrop 2000 detection showed that the concentration of the RNA was above 180 ng/μL and that the OD260/280 was between 1.80 and 2.01. Electrophoretic detection showed that the RNA bands of the samples were clear and complete, without obvious tailing and degradation, and that there were no DNA bands; the total RNA extracted from the skin was of good quality and met the requirements of the test. At three key timepoints in cashmere growth, the relative expressions of the candidate genes in the skin of long-haired and short-haired cashmere goats were determined, an analysis of variance was carried out, a statistical table was created (Table 4), and a drawing was made by GraphPad Prism 8.3.0 software (Figure 7).

Only in March was the expression of *KRT25* in LHGs significantly higher than that in SHGs (*p* < 0.05), but it was found that the expression of LHGs was higher than that of SHGs. The expression of *KRT27* in LHGs was significantly higher than that in SHGs in March (*p* < 0.05), but there was no significant difference in September and December. The difference in *KRT39* between LHGs and SHGs was significant in March and December (*p* < 0.05), and was extremely significant in September (*p* < 0.01). The expression of LHGs was higher than that of SHGs, and the difference was about 1.7 times. The expression of *KRT74* in LHGs was significantly higher than that in SHGs in September, December, and March (*p* < 0.05), and the difference was about 1.6 times. The difference in *LOC100861184* between LHGs and SHGs was significant in March and December (*p* < 0.05), and was extremely significant in September (*p* < 0.01). The expression of LHGs was higher than that of SHGs, and the difference was about 1.8 times. The expression of *LOC102177231* in LHGs was extremely significantly higher than that in SHGs only in 3 months (*p* < 0.01), but there was no significant difference in other periods, and it was found that the expression of LHGs was higher than that of SHGs. The expression of *LOC102178767* in LHGs was significantly higher than that in SHGs in September (*p* < 0.05), December, and March, and the difference was about two times. The expression of *LOC102179881* in LHGs was extremely significantly higher than that in SHGs in September and March (*p* < 0.01), and was significantly higher in LHGs than in SHGs in December (*p* < 0.05); the difference was about three times. The expression of *LOC106503203* in LHGs was extremely significantly higher than that in SHGs in September and December (*p* < 0.01), and in March the expression level of LHGs was significantly higher than that in SHGs (*p* < 0.05); the difference was about 2.3 times. The expression of *LOC108638293* in LHGs was extremely significantly higher than that in SHGs in September and March (*p* < 0.01). The expression of *LOC108638298* in LHGs was significantly higher than that in SHGs in September and March (*p* < 0.05), and was extremely significantly higher in LHGs than in SHGs in December (*p* < 0.01).

### 3.6. Correlation Analysis between Gene Expression and Hair Length 

The correlation analysis between the relative expression of candidate gene mRNA in skin tissue and hair length traits in Inner Mongolia cashmere goats is shown in Table 5. The results showed that the relative expression of the *KRT39*, *KRT74*, *LOC100861184*, *LOC102177231*, *LOC102178767*, *LOC102179881*, *LOC106503203*, *LOC108638293*, and *LOC108638298* genes showed a very significant positive correlation with hair length. The Pearson correlation coefficient was high, ranging from 0.59 to 0.89. There was no significant correlation between the relative expression of the *KRT25* and *KRT27* genes with hair length.

## 4. Discussion

A WGCNA is a systematic genetic analysis method, which is suitable for the study of some complex traits. In this study, the RNA-seq and phenotypic data of different hair types of Inner Mongolia cashmere goats for 12 months were used for the WGCNA. According to the module division of 7320 differential genes, 19 coexpression modules were obtained. It was found that the genes in the magenta module might be related to different hair types in Inner Mongolia cashmere goats.

The GO function of this module gene is mainly enriched in the cellular components, which are mainly involved in intermediate filaments (GO:0005882), intermediate filament cytoskeletons (GO:0045111), and cytoskeletal parts (GO:0044430). It was found that this module contains a large number of KRTs and KRTAPs. As shown in Figure 5, most of the genes in the network are involved in intermediate filaments. Intermediate filaments are a kind of cytoskeleton structure that form a unique slender structure, which is characterized by being 10 nm in diameter and occurring in the cytoplasm of eukaryotic cells. The intermediate filaments form a fiber system, which is composed of chemical heterogeneous subunits and participates in the mechanical integration of various components of the cytoplasmic space. At the same time, the structure of hair and cashmere fiber is basically composed of KTRs and KRTAPs [35,36,37]. The composition and content of keratin are closely related to the quality of cashmere fiber, so the study of KRTs and KRTAPs can explain the mechanism of the formation of villus quality traits at the molecular level [38]. KRTs and KRTAPs are essential components of hair growth. In this study, it was found that the expressions of this module’s genes are generally high in LHGs, which may be due to the fact that LHGs have longer hair and need to express a large number of genes and proteins related to hair composition.

From Figure 4A, we can find that most of the genes in the magenta module express the same between April and October, but there is a significant difference between November and March. It is speculated that November to March may be the key period for the difference between the two hair types. The period from November to March is a cold winter, and the decrease in the environmental temperature and the shortening of light time may affect the growth of hair. From Figure 6A–H, we can see that the candidate genes begin in the middle of anagen (June–November), and the gene expression increases gradually, reaching the highest level in October–November. During catagen, telogen, and early anagen (December–June), the gene expression began to decrease gradually. The gene expression levels of the two hair types were significantly different from December to June, and it could be seen that the gene expression level of the long-hair type decreased slowly during catagen and telogen (December–March). The gene expression level decreased rapidly in early anagen (April–June). On the other hand, the gene expression level of the short-hair type began to decrease rapidly in catagen and telogen (December–March), and was relatively stable in early anagen (April–June).

Many studies have shown that KRTs and KRTAPs are associated with a variety of hair traits, including fiber bending, fiber fineness, fiber length, color, and the hair follicle cycle. *KRT25*, a member of the keratin family, belongs to type I inner root sheath keratin, which is specifically expressed in the inner root sheath, mainly in the medulla, premedulla, Huxley layer, Henle layer, and stratum corneum of the inner root sheath. Type I inner root sheath keratin includes *KRT25*, *KRT26*, *KRT27*, and *KRT28*; type II inner root sheath keratin includes *KRT71*, *KRT72*, *KRT73*, and *KRT74*. These proteins are specifically expressed in the inner root sheath of hair follicles [39]. 

*KRT25* plays an important role in some human hair diseases. Autosomal recessive woolly hair (ARWH) is a relatively rare hereditary hair disease characterized by sparse, short, and curly hair. This disease is caused by *KRT25* mutations [40]. The homozygous missense variation in the *KRT25* (c.950T > C, p.Leu317Pro) may lead to the destruction of the protein structure, which may interfere with the heterodimerization of *KRT25* and type II keratin in the root sheath of hair follicles and in the medulla of the hair stem, resulting in ARWH, indicating that the expression of *KRT25* will affect the structure and growth of hair [41]. Kang [42] found that *KRT25*, *KRT5*, *KRT71*, and *KRT14*, members of the keratin gene family, may be related to the hair curls of Tan sheep. In this study, it was also found that *KRT25* may be associated with the hair growth rate, and that the expression of *KRT25* in LHGs was significantly higher than that in SHGs.

*LOC102177231* (*KRT71*) and *KRT74* belong to type II inner root sheath keratin, both of which come from goat chromosome 5. Previous studies have confirmed that mutations in the *KRT71* and *KRT75* genes are associated with autosomal dominant woolly hair (ADWH). ADWH often occurs before the age of 2 years. ADWH is characterized by slow hair growth, a gray color, fragile hair fibers, tight curls, a wool-like appearance, and diffuse sparsity [38,43]. Several studies have found that there is a missense variation in exon 2 of the *KRT71* gene (C.451C > T), and mutations in exon 7 (c.1266_1273delinsACA) can regulate hair curls [42,44,45]. In this study, it was found that the expression content of the *LOC102177231* gene in LHGs was higher than that in SHGs, and there was a significant positive correlation between the expression of this gene and hair length.

*KRT74* belongs to type II epithelial keratin, also known as *K6irs4*, which is an intermediate filament protein responsible for the structural integrity of epithelial cells. It is specifically expressed in the inner root sheath and plays a role in hair formation. Type II epithelial keratin K74, encoded by the *KRT74* gene, is heavily expressed in the Hershley layer of the root sheath in hair follicles, which plays an important role in maintaining hair growth and stabilizing hair morphology [46]. Shimomura [47] studied a Pakistani ADWH family and found a heterozygous mutation (c.444 C > G) in exon 1 of the *KRT74* gene, which destroys the formation of the intermediate filaments of K74 keratin, thus affecting hair growth. In this study, we explored the difference in *KRT74* gene expression in cashmere goat skin. It was found that the expression of the *KRT74* gene in LHGs was significantly higher than that in SHG. Through correlation analysis, it was found that the expression of the *KRT74* gene was positively correlated with wool length. It can be inferred that the expression of the *KRT74* gene can promote the growth of hair length and regulate other cashmere quality traits.

Some studies have found that the expression of KRT27 decreases gradually during catagen and stops at telogen, which plays an important role in the maintenance of hair follicle morphology and fiber morphology [48]. In this study, no significant difference was found in the hair follicle cycle, but the expression of the LHG was significantly lower than that of the SHG during telogen.

*KRT33A* is a type I hair keratin, which is expressed only in the cortex of hair fibers [49]. Some studies have confirmed that *KRT33A* is located in the cortex part of cashmere fiber and is a structural protein in goat villi fiber, and it is found that *KRT33A* is highly expressed in winter [50]. It was found that the expression of *LOC102179881* (*KRT33A*) in the LHG was higher than that in the SHG in the three stages, but there was no difference in the cycle. The expression of the SHG was higher in December than in other months. The *KRT39* gene belongs to type I keratin and has been found to regulate the periodic development of hair follicles in yaks [51]. It was found that the *KRT39* gene plays a potentially important role in regulating the fineness of cashmere in Tibetan cashmere goats [52]. In this study, it was found that the *KRT39* gene was differentially expressed in the skin of LHGs and SHGs. The expression level of LHGs was significantly higher than that of SHGs.

Some studies have found that the upregulation of KRTAP4 isoform genes’ expression can lead to white hair. The expression of KRTAP4 isoform genes (*KRTAP4-8*, *KRTAP4-9*, etc.) in white hair is significantly higher than that in black hair [53]. In Merino sheep, the expression of *KRTAP4-3* was different between black and white hair [54]. At the same time, the expression level of KRTAP4 isoform genes in straight hair was higher than that in curly hair [55]. This study also found that KRTAP4 isoform genes (*LOC102178767*, *LOC106503203*) can regulate hair growth length.

## 5. Conclusions

In conclusion, through a WGCNA of gene expression data of different hair types in Inner Mongolia cashmere goats, a total of 19 coexpression modules were obtained. It was found that there was a strong correlation between the magenta module and different hair types. It was found that the expression trends of genes in the magenta module were different between the two hair types, with high expression in LHGs and low expression in SHGs. GO functions are mainly concentrated in cellular components, including intermediate filaments (GO:0005882), intermediate filament cytoskeletons (GO:0045111), and cytoskeletal parts (GO:0044430). The KEGG pathway is mainly enriched in arginine as well as proline metabolism (chx00330) and the MAPK signaling pathway (chx04010). The candidate genes of different hair types, including the *KRT39*, *KRT74*, *LOC100861184*, *LOC102177231*, *LOC102178767*, *LOC102179881*, *LOC106503203*, *LOC108638293,* and *LOC108638298* genes, were screened. Through qRT-PCR, it was found that there were significant differences in these candidate genes between the two hair types, and most of them had a significant positive correlation with hair length. It was preliminarily inferred that these candidate genes could regulate different hair types of cashmere goats and provide molecular markers for hair growth.

## Figures and Tables

**Table 1:** Primer sequences of candidate genes and reference genes.

Gene Name	Primer Sequences		Product Length (bp)	TM (°C)
*KRT25*	F:	AAGTCAGGACCGAGACCGAG	129	61
	R:	CAGACTTGCAAGCCCCATCAT		
*KRT27*	F:	ACTTCACGGTAATTGACGACCT	283	60
	R:	GCCTTCATCTCCTCCTCATGG		
*KRT39*	F:	TGAGATTGCCACATACCGCA	169	59
	R:	CTCATGTATCCCACAGGGGC		
*KRT74*	F:	GATCAATCAGCGCACAGCAG	152	60
	R:	CCACCTCCGCATCGTACAAA		
*LOC102179881*	F:	GTGCAGAGCCTGATCGTCAA	252	60
	R:	TCCACACCGAGTACGTGAGA		
*LOC108638298*	F:	GCGCTCACCATCCCTAAGTA	117	60
	R:	GGGTGCCTCCTAGTTTGAAGA		
*LOC102177231*	F:	TGTGGATGCTGCTTATGCCA	171	60
	R:	AGGTTTAGGTCGCGGTTGTT		
*LOC100861184*	F:	CCCTGTGTACTGCCACAGAA	198	60
	R:	TGACAGGAGGATCAGCAGGA		
*LOC108638293*	F:	GCCAAGCCCAGGAATGT	219	60
	R:	CCAGTTGTCAGCAAAGTCTC		
*LOC106503203*	F:	AAACTCACCCAGAACCTCCA	112	58
	R:	GGACGGTAGCAGGTCTCTT		
*LOC102178767*	F:	GTCTCCTGCCACACCACTTG	164	60
	R:	CCTAAGGGTCAGCGCGAAA		
*GAPDH*	F:	GCAAGTTCCACGGCACAG	118	60
	R:	TCAGCACCAGCATCACCC		
*β-actin*	F:	GGCAGGTCATCACCATCGG	158	60
	R:	CGTGTTGGCGTAGAGGTCTTT		

**Table 2:** Statistical table of modules’ gene numbers.

Module Color	Gene Numbers	Module Color	Gene Numbers
Black	181	Magenta	128
Blue	1839	Midnight blue	90
Brown	620	Pink	168
Cyan	92	Purple	110
Green	343	Red	324
Green-yellow	102	Salmon	96
Grey	254	Tan	102
Grey60	68	Turquoise	2207
Light cyan	79	Yellow	469
Light green	48		

**Table 3:** Candidate gene information table.

Gene ID	Gene Name	Gene Description
102178766	*KRT9*	Keratin 9
100861172	*KRT25*	Keratin 25
100861382	*KRT27*	Keratin 27
102178207	*KRT39*	Keratin 39
102176789	*KRT74*	Keratin 74
100861180	*KRTAP3-1*	Keratin-associated protein 3-1
100861173	*KRTAP11-1*	Keratin-associated protein 11-1
100861184	*LOC100861184*	Keratin-associated protein 9.2
102177231	*LOC102177231*	Keratin, type II cytoskeletal 71
102178767	*LOC102178767*	Keratin-associated protein 4-8-like
102179881	*LOC102179881*	Keratin, type I microfibrillar, 47.6 kDa, *KRT33A*
106503203	*LOC106503203*	Keratin-associated protein 4-9-like
108638293	*LOC108638293*	Keratin-associated protein 9-9-like
108638298	*LOC108638298*	Keratin, high-sulfur matrix protein, IIIA3-like

**Table 4:** Statistical table of the relative expression of the candidate genes.

Hair Type	Gene Name	Sep.	Dec.	Mar.	Gene Name	Sep.	Dec.	Mar.
LHG	*KRT25*	4.16 ± 1.68	5.42 ± 1.04	5.54 ± 1.52	*LOC102178767*	6.63 ± 1.79	6.72 ± 1.25	9.14 ± 6.79
SHG		3.98 ± 0.59	4.24 ± 1.65	3.23 ± 1.18		4.31 ± 1.59	4.86 ± 1.39	2.15 ± 1.18
*p*-value		0.0124	0.8059	0.1633		0.0208	0.0199	0.0269
LHG	*KRT27*	7.08 ± 3.40	6.69 ± 3.43	9.28 ± 2.77	*LOC102179881*	19.11 ± 9.17	20.30 ± 8.60	12.81 ± 4.78
SHG		6.20 ± 1.79	7.50 ± 4.12	6.01 ± 2.88		5.91 ± 2.30	8.41 ± 4.74	3.23 ± 1.41
*p*-value		0.4539	0.4878	0.0335		0.0062	0.017	0.0004
LHG	*KRT39*	7.20 ± 1.85	7.40 ± 1.88	6.65 ± 2.75	*LOC106503203*	30.11 ± 4.94	18.31 ± 6.01	25.26 ± 17.80
SHG		4.00 ± 0.73	4.79 ± 1.82	3.79 ± 0.78		19.21 ± 7.92	8.70 ± 3.70	4.65 ± 1.72
*p*-value		0.03	0.0019	0.0357		0.0052	0.0054	0.0387
LHG	*KRT74*	5.41 ± 1.14	5.54 ± 1.75	4.41 ± 1.73	*LOC108638293*	40.52 ± 17.15	32.94 ± 16.17	30.71 ± 20.37
SHG		3.58 ± 1.12	3.42 ± 1.16	2.69 ± 0.66		21.17 ± 7.44	33.81 ± 9.12	5.58 ± 4.20
*p*-value		0.0416	0.0159	0.0291		0.0194	0.6067	0.0144
LHG	*LOC100861184*	7.19 ± 2.10	6.69 ± 2.79	5.22 ± 3.02	*LOC108638298*	12.57 ± 4.99	10.12 ± 2.78	14.17 ± 12.02
SHG		3.80 ± 1.82	3.22 ± 0.99	3.86 ± 2.85		6.51 ± 1.41	3.79 ± 1.05	2.75 ± 1.16
*p*-value		0.0075	0.0131	0.037		0.0124	0.0002	0.0371
LHG	*LOC102177231*	10.72 ± 5.43	7.16 ± 3.04	11.08 ± 5.28				
SHG		9.63 ± 3.40	5.38 ± 2.55	3.54 ± 1.65				
*p*-value		0.4687	0.448	0.0068				

**Table 5:** Correlation analysis between the expression of genes’ mRNA and hair length traits.

Gene Name	Correlation Coefficient between Hair Length and mRNA Expression	*p*-Value
*KRT25*	0.5066	0.0645
*KRT27*	0.4060	0.1498
*KRT39*	0.81085	0.0004
*KRT74*	0.76605	0.0014
*LOC100861184*	0.79705	0.0006
*LOC102177231*	0.59344	0.0253
*LOC102178767*	0.81826	0.0003
*LOC102179881*	0.88643	<0.0001
*LOC106503203*	0.8966	<0.0001
*LOC108638293*	0.63469	0.0148
*LOC108638298*	0.73014	0.003

## Data Availability

The transcriptome dataset used in this study can be found in the SRA database and is expected to be made public on 30 June 2024 with the login number PRJNA832904.

## Supplementary Materials

The following supporting information can be downloaded at: https://www.mdpi.com/article/10.3390/ani12111456/s1, Supplemental Table S1: Magenta module gene information statistical table; Supplemental Table S2: KEGG enrichment analysis table of the magenta module; Supplemental Table S3: GO analysis table of the magenta module.
